# Supplementary material for: Paired Exome-Based Comprehensive Genomic Profiling and Germline Genetic Testing for Unselected Patients With Colorectal Cancer in a Multicenter Prospective Study
Source: Gastro Hep Adv. 2026 Aug 12;5(11):101093. doi: 10.1016/j.gastha.2026.101093 (PMC13572061; doi:10.1016/j.gastha.2026.101093)
Supplement: Supplementary file 1 [file mmc1.pdf]

Gastro Hep Advances 2026;5:101093

ORIGINAL RESEARCH—CLINICAL

Paired Exome-Based Comprehensive Genomic Profiling and Germline Genetic Testing for Unselected Patients With Colorectal Cancer in a Multicenter Prospective Study

Pedro Luiz Serrano Uson, Jr,[1,2,*] Jacquelyn Reuther,[3,*] Michael A. Golafshar,[4] Megan H. Hawley,[3] Emily Lavy,[3] Sarah M. Young,[3] Tanios Bekaii-Saab,[1] Christina Wu,[1] W. Michael Korn,[3,§] Edward D. Esplin,[3,5,§] Jianhua Zhao,[3] Katie L. Kunze,[4] Robert Daber,[3] and Jewel Samadder[1]

[1]*Mayo Clinic Cancer Center, Phoenix, Arizona;* [2]*Center for Personalized Medicine, Hospital Israelita Albert Einstein, São Paulo, Brazil;* [3]*LabCorp (formerly Invitae Corp.), San Francisco, California;* [4]*Quantitative Health Sciences, Scottsdale, Arizona; and* [5]*Stanford University, Stanford, California*

BACKGROUND AND AIMS: Comprehensive genomic profiling (CGP) for tumors and germline genetic testing (GGT) inform precision therapy and clinical management of patients with colorectal cancer (CRC), and evidence is growing in support of universal paired CGP-GGT patient testing. However, the utility of combining CGP and GGT for early-stage CRC (ESC) and early-onset CRC (EOC) is unclear. **METHODS:** We performed a prospective, multisite study featuring GGT using an 80+ gene next-generation sequencing platform and exome-based CGP among CRC patients (unselected for age, stage, family history) receiving care at Mayo Clinic Cancer Centers between April 1, 2018, and March 31, 2020. **RESULTS:** A total of 150 CRC patients had GGT and exome-based CGP performed. ESC patients had an enrichment of high microsatellite instability and high tumor mutation burden. High microsatellite instability was also enriched in those with smoking history, and in tumors with mutated *BRAF*, homologous recombination deficiency, or at least 1 variant in the rat sarcoma virus pathway. Moreover, patients with smoking history were enriched in *BRAF* and other Tier 1 or 2 variants overall. Sixteen percent of patients harbored a pathogenic germline variant, most frequent being in Lynch syndrome genes. Paired GGT and CGP testing had high rates of clinically significant findings (≈70%) with the most frequent being high tumor mutation burden status. Pathway and mutational signature analysis revealed frequent CGP mutations in DNA repair and cell cycle pathways. **CONCLUSION:** These data suggest that universal, combined GGT-CGP increases clinical utility for EOC and ESC patients. This is key for EOC patients who tend to experience poorer outcomes. CGP-GGT expedites germline resolution for tumor mutations in hereditary cancer genes, reducing delays and facilitating identification of relevant therapies, clinical trials, and management recommendations.

Keywords: Genetic Testing; Colorectal Cancer; Lynch Syndrome; Microsatellite Instability

Introduction

Colorectal cancer (CRC) is the third most common cancer worldwide, accounting for about 10% of all cancer cases, and is the second leading cause of cancer-related deaths.[1] In 2022, there were an estimated 1,926,118 new cases and 903,859 deaths of CRC worldwide.[1] In the United States, in 2024, there were an estimated 152,810 new cases and 53,010 deaths.[2]

Risk factors for developing CRC include age, family and personal history of colon or related cancers, and lifestyle factors.[2,3] About 15% of patients with CRC are carriers of pathogenic germline variants (PGVs), most in moderate- and high-penetrance cancer susceptibility genes.[4]

Early-onset CRC (EOC) is on the rise.[3] EOC is usually defined as a patient that develops CRC before 50 years old.[3] The definition of the age of EOC is based on historical screening indications and could change in face of new screening guidelines recommendations (ie, 45 years old).[3] Multiple factors are related to increased incidence of CRC in young patients worldwide, which would be related to PGVs, polygenic and familial risk, environmental exposures (exposome), and even a high-risk microbiota related to Western diets.[3]

Paired germline and somatic analysis in patients with CRC are valuable to not only identify the full scope of genomic alterations but also as a strategy to maximize actionable treatment for precision medicine initiatives and genetic counseling, in both late-onset and EOC.[5]

*Denotes co-first authorship.
[§]Previous affiliation.

Abbreviations used in this paper: CGP, comprehensive genomic profiling; CI, confidence interval; CNV, copy number variant; CRC, colorectal cancer; dMMR, defective mismatch repair; EOC, early-onset CRC; ESC, early-stage CRC; GGT, germline genetic testing; HRD, homologous recombination deficiency; HRR, homologous recombination repair; MMR, mismatch repair; MSI, microsatellite instability; MSI-H, high microsatellite instability; MSS, microsatellite stable; NGS, next-generation sequencing; OR, odds ratio; PGV, pathogenic germline variant; SNV, single nucleotide variant; TMB, tumor mutation burden; TMB-H, high tumor mutation burden.

Most current article

© 2026 The Authors. Published by Elsevier Inc. on behalf of American Gastroenterological Association Institute. This is an open access article under the CC BY license (http://creativecommons.org/licenses/by/4.0/).
2772-5723
https://doi.org/10.1016/j.gastha.2026.101093

However, barriers have been identified with implementation of paired universal testing, including lack of consensus on how to integrate the findings, lack of guidelines, insurance coverage, concern about potentially widening health inequities, potential liability or clinician hesitancy, and the real-world actionability of the findings.[5–7]

With the objective of evaluating the impact of universal testing for late and EOC (<45 y/o), prospective exome-based comprehensive genomic profiling (CGP) on tumor samples and germline genetic testing (GGT) were performed for a cohort of patients with CRC.

Materials and Methods

Colorectal Cancer Cohort

A prospective unselected CRC patient cohort (n = 150) of patients receiving care at Mayo Clinic Cancer Centers in Rochester, Minnesota; Jacksonville, Florida; and Phoenix, Arizona, and in a community oncology practice (Mayo Clinic Health System, Eau Claire, Wisconsin), between April 1, 2018, and March 31, 2020, for the Interrogating Cancer Etiology Using Proactive Genetic Testing program was assessed by paired GGT and CGP.[7] CGP included assessment of sequence variants, amplifications, microsatellite instability (MSI) and tumor mutation burden (TMB). The study methodology for Interrogating Cancer Etiology Using Proactive Genetic Testing is briefly described below, and additional details are available in Samadder et al.[7] Research coordinators completed patient recruitment using central lists of daily oncology clinic visits. Patients were unselected for race/ethnicity, disease stage, family or personal history of cancer, age at disease onset, and other parameters. All patients viewed a standardized pretest genetics education video, were offered pre-test genetic counseling, and provided written informed consent. Data were deidentified to all except study investigators. Detailed clinicopathological features were collected from medical records, self-administered electronic questionnaires, and an electronic family cancer pedigree tool (Cancer Gene Connect). This study was approved by the Mayo Clinic Institutional Review Board (19–010300). EOC was defined as onset of CRC <45 years of age. Early-stage CRC (ESC) was defined as stages 0-III.

DNA Isolation and Sequencing

Specimens underwent clinical testing at Invitae (now part of LabCorp San Francisco, CA), a Clinical Laboratory Improvement Amendments-certified, New York state-certified and College of American Pathology–accredited genetic diagnostics laboratory. Genomic DNA for GGT was isolated from whole blood and saliva using a Hamilton STAR (Hamilton Company, Reno, NV) and the Omega Bio-tek HDQ DNA extraction kit (Omega Bio-tek, Norcross, GA). Next-generation sequencing (NGS) enriched targeted regions using a hybridization-based protocol and sequenced using Illumina technology (Illumina; La Jolla, CA). Hybridization capture probes targeted 80 or more genes, featuring full-gene sequencing (including coding exons, 20 base pairs of adjacent intronic sequencing on either side of the coding exons and select noncoding variants) and deletion-duplication analysis for single nucleotide variants (SNVs), small insertion/deletions (indels), copy number variants (CNVs), and splice site variants. All targeted regions were sequenced with ≥50x depth or were supplemented with additional analysis.[8] Confirmation of the presence and location of reportable variants is performed as needed based on stringent criteria using one of several validated orthogonal approaches.[9]

DNA was extracted from formalin-fixed paraffin-embedded tumor tissue for CGP using the DNA formalin-fixed paraffin-embedded isolation system (Promega) on the automated King Fisher Flex platform (Thermo Fisher). NGS libraries of the tumor DNA were generated using the DNA Library Prep Kit with Fragmentation (Watchmaker Genomics; Boulder, CO) and underwent exome capture using custom probes (targeting coding exons, 10 base pairs of adjacent intronic sequencing on either side of the coding exons and select noncoding variants) with the Fast Hybridization and Wash Kit with Amplification Mix (Twist Bioscience; San Francisco, CA). The sequencing was performed on an Illumina NovaSeq6000 using 2 × 150 reads, to at least 250x mean coverage (range 260–1189x).

Germline Specimen Processing and Review

Full gene sequencing, deletion/duplication analysis, and variant interpretation using an NGS panel of 80+ genes on the Invitae Multi-Cancer panel was offered at no cost as previously described.[7] NGS reads were aligned to the reference human genome sequence hg19 (GRCh37) using NoVo align (NoVo craft Technologies; Selangor, Malaysia). Small indels and SNVs were analyzed using the Genome Analysis Toolkit Unified Genotyper,[10] Freebayes,[11] and Coalgen (unpublished). CNV calls were performed using the in-house algorithm, CNVitae.[12] Large structural variants were detected using split-read analysis as described previously.[13] More details regarding workflow are described previously.[8,9] The technique was used at all *loci*, allowing for the detection of certain structural variants that can evade both read-depth analysis (those with breakpoints within a small assay target) and indel detection (large structural variants or with breakpoints outside of targeted regions). Variants were assessed using a refined version of the American College of Medical Genetics and Genomics criteria (Sherloc).[14] Alterations were defined as on-target when they occurred in genes associated with hereditary cancer syndrome with an associated increased risk for CRC; while alterations were considered as off-target when they occurred in hereditary cancer risk syndrome genes not commonly associated with an increased risk for CRC.

Tumor Specimen Processing and Review

Tumor sequencing FASTQs were preprocessed by fastp (v0.23.4)[15] and were aligned to the hg19 human genome by BWA (v0.7.17)[16] and Abra2 (v2.2).[17] PCR duplicates were removed by Picard (v3.1.1) (http://broadinstitute.github.io/picard). SNVs and Indels were detected by Vardict (v1.8.3)[18] and MUTECT2 (GATK4 v.4.2.0.0).[19] Variants were annotated using Nirvana Annotator (v0.3).[20] Variants were assessed according to Association of American Pathology/American Society of Clinical Oncology/College of American Pathology guidelines[21] with review limited to variants occurring in 191 clinically significant cancer-associated genes. MSI was assessed utilizing a custom tool, AbsoluteMSI (v0.1) (unpublished) and reported as microsatellite stable (MSS) or unstable (high microsatellite instability [MSI-H]). CNV profile was detected by

PureCN (v0.3)[22] and manually reviewed for gene level amplifications for select clinically significant genes (*ERBB2, FGFR1-3, CCND1, CCNDE, MYC*) as well as genes mutated in the germline or somatic sample in which a possible second hit is potentially indicated. Nonsynonymous exonic variants that exhibited a high posterior probability of being somatic after assessment by somatic-germline-zygosity algorithm were utilized to calculate TMB.[23] Somatic findings were further assessed for clinical significance based on treatment associations in CRC, treatment associations in other solid tumors, prognostic implications for CRC, and clinical trial eligibility. Tumor samples were assessed for quality control metrics based on variant-type based thresholds. All samples passed SNV/small-indel variant quality control (QC) while 13 failed MSI QC and 16 failed TMB QC; variant types failing QC were excluded from analysis. On-target variations were defined as changes in the DNA that occurred at the specific and intended location of genetic locus; off-target variations were variations that occurred at unintended locations of the genome. Tier 1 and tier 2 variations were defined as clinical and potential meaningful variants, related to significance, actionability, and evidence of impact of patient care.

Statistical Assessments and Figure Generation

Patient demographics and genetic results were compiled and assessed in R (https://www.R-project.org) to produce summary, Pearson Chi-squared test, Fisher exact test, figures, and additional assessments.[24] Oncoprinter was utilized for the co-occurrence assessment.[25,26] Pathway analysis was completed using Reactome GSA.[27] Mutational signatures were assessed with mut Signatures R package.[28]

Results

Cohort Characteristics

General patient demographics and disease characteristics are shown in Table. Forty-two percent (63/150) of patients were female and 84.0% (126/150) were non-Hispanic White. EOC was present in 19.3% (29/150) of patients. Sixty-two percent (18/29) of patients with EOC were diagnosed at an early stage (0 to III). Late-stage disease was diagnosed with 30.0% (45/150) of patients. The most common tumor location was rectal in 43.3% (65/150) of patients, and surgical resection was performed in 91.3% (137/150) of cases. Comorbidities included a history of smoking in 39.3% (59/150), obesity in 37.3% (56/150), hypertension in 38.0% (57/150), and diabetes in 8.0% (12/150) of patients. A family history of cancer was reported in 60.0% of patients (90/150), and a family history of gastrointestinal cancers was reported in 24.7% (37/150) of patients.

Germline Analysis

For the 150 CRC patients included in this study, PGVs were detected in 24/150 patients (16.0%), 58 (38.7%) had uncertain results, and 68 (45.3%) had negative results. Of PGV results, 45.8% (n = 11) were on-target and 54.2% (n = 13) were off-target. The most common on-target PGVs were in Lynch syndrome genes (*MSH2*, n = 4; *MSH6*, n = 1; *MSH3*, n = 1). Logistic regression modeling showed family history of gastrointestinal cancers (odds ratio [OR] = 2.62, 95% confidence interval [CI] = 1.03–6.53, P = .039) and obesity (OR = 0.12, 95% CI = 0.02–0.44, P = .006) were associated with having a PGV result. Fourteen cases were considered to have clinically significant germline findings with defined clinical management guidelines (9.3%). Of these, 28.6% (4/14) had precision therapy approved in another tumor type (ie, Poly Adenosine Diphosphate Ribose Polymerase inhibitors), and 64.3% (9/14) with potential clinical trials eligibility (Supplementary Table 1).

Somatic Analysis

Variant analysis. Of the 150 patients, 132 (88.0%) had somatic findings of established or potential clinical significance (Tier 1/Tier 2), including detection of 707 total Tier 1 or Tier 2 variants (Figure 1). Patients had a median of 4 Tier 1 or Tier 2 variants (Figure 1C) with missense and loss of function variants, particularly nonsense and frameshift deletions (Figure 1B) being the most common type of alterations. By logistic regression, colon cancers were significantly associated with a lower likelihood of somatic findings when compared to rectal cancers (OR 0.12, 95% CI 0.02–0.39, P = .001). The most frequent somatic findings were *APC* loss of function variants and *TP53* hotspot missense variants (Figure 1). Half of patients with *APC* variants harbored more than 1 variant. Additionally, the *APC* c.835-8A>G splice variant was detected in 4.7% (n = 7) of patients (Supplementary Figure 1). Multiple variants were found to be recurrent in the cohort including *KRAS* c.35G>T (p. Gly12Val) and c.38G>A (p. Gly13Asp), *BRAF* c.1799T>A (p. Val600Glu), *RNF43* c.1976del (p. Gly659ValfsTer41), and *TCF7L2* c.1385dup (p. Cys463ValfsTer8) (Supplementary Figure 1). Additionally, BRAF mutations were found to be statistically associated with smoking history (P = .027). The somatic mutational spectrum did not statistically differ between EOC and typical onset CRC. While not statistically significant, select gene variants were observed at lower rates in the EOC patients, including *BRAF, RNF43, MSH3, BLM, AMER1, ATM*, and *CREBBP*. Variants of established significance were concentrated in *KRAS, BRAF*, and mismatch repair (MMR) genes (*MSH6* and *MSH3*). In 77 patients (51.3%), 129 Tier 1/2 variants were detected in hereditary cancer syndrome associated genes (n = 26) at allele frequencies of 30.0%–70.0%, suggesting possible germline origin. Of those, 18/129 (14.0%) were confirmed to be germline variants. When considering only genes where variants have high germline likelihood (*BLM, MSH6, CHEK2, MSH2, AXIN2, MLH1, BARD1, ATM, BRCA2, RAD51D, BRCA1, MSH3, FLCN, SDHD, BRIP1, MUTYH, SDHA*), when detected somatically, 16/34 (47.1%) were confirmed to be germline variants.

Tumor Mutation Burden/Microsatellite Instability

TMB ranged from 0.5 to 245.9 mutations/megabase (muts/Mb) with a mean of 15.5 muts/Mb. Twenty-seven samples (18.0%) had a TMB ≥10 muts/Mb (high tumor mutation burden [TMB-H]). Characterization of MSI

Table. Patient Demographics

Characteristics	<45 (N = 29)	≥45 (N = 121)	Total (N = 150)	*P* value
Germline result				.443[a]
Positive	6 (20.7%)	18 (14.9%)	24 (16.0%)	
VUS/negative	23 (79.3%)	103 (85.1%)	126 (84.0%)	
Somatic result				.760[a]
Positive	26 (89.7%)	106 (87.6%)	132 (88.0%)	
Negative	3 (10.3%)	15 (12.4%)	18 (12.0%)	
Somatic mutation in hereditary cancer gene (germline negative cases only)				.141[a]
Yes	8 (72.7%)	43 (89.6%)	51 (86.4%)	
No	3 (27.3%)	5 (10.4%)	8 (13.6%)	
Missing	18	73	91	
Germline/somatic status				.368[a]
Germline and somatic	6 (23.1%)	15 (13.8%)	21 (15.6%)	
Germline only	0 (0.0%)	3 (2.8%)	3 (2.2%)	
Somatic only	20 (76.9%)	91 (83.5%)	111 (82.2%)	
No germline or somatic variants	3	12	15	
Age at Dx				<.001[b]
Mean (SD)	39.9 (4.9)	59.9 (9.8)	56.0 (12.0)	
Median	42.0	61.0	56.0	
Range	27.0–44.0	45.0–79.0	27.0–79.0	
Gender				.237[a]
Male	14 (48.3%)	73 (60.3%)	87 (58.0%)	
Female	15 (51.7%)	48 (39.7%)	63 (42.0%)	
Race				.718[a]
White	25 (86.2%)	101 (83.5%)	126 (84.0%)	
Non-White	4 (13.8%)	20 (16.5%)	24 (16.0%)	
Ethnicity				.615[a]
Hispanic/Latino	1 (3.4%)	7 (5.8%)	8 (5.3%)	
Non-Hispanic	28 (96.6%)	114 (94.2%)	142 (94.7%)	
Smoking				.022[a]
Yes	6 (20.7%)	53 (43.8%)	59 (39.3%)	
No	23 (79.3%)	68 (56.2%)	91 (60.7%)	
Body mass index >30				.435[a]
Yes	9 (31.0%)	47 (38.8%)	56 (37.3%)	
No	20 (69.0%)	74 (61.2%)	94 (62.7%)	
Diabetes mellitus				.077[a]
Yes	0 (0.0%)	12 (9.9%)	12 (8.0%)	
No	29 (100.0%)	109 (90.1%)	138 (92.0%)	
Hypertension				.003[a]
Yes	4 (13.8%)	53 (43.8%)	57 (38.0%)	
No	25 (86.2%)	68 (56.2%)	93 (62.0%)	
Localization				.354[a]
Left sided	6 (20.7%)	35 (28.9%)	41 (27.3%)	
Rectum	16 (55.2%)	49 (40.5%)	65 (43.3%)	
Right sided	7 (24.1%)	37 (30.6%)	44 (29.3%)	
AJCC 8th stage at diagnosis				.233[a]
Stage I/II	5 (17.2%)	40 (33.1%)	45 (30.0%)	
Stage III	13 (44.8%)	47 (38.8%)	60 (40.0%)	
Stage IV	11 (37.9%)	34 (28.1%)	45 (30.0%)	
Surgery				.721[a]
Yes	26 (89.7%)	111 (91.7%)	137 (91.3%)	
No	3 (10.3%)	10 (8.3%)	13 (8.7%)	
CEA (low/high)				.476[a]
>5	10 (37.0%)	36 (30.0%)	46 (31.3%)	
≤5	17 (63.0%)	84 (70.0%)	101 (68.7%)	
Missing	2	1	3	
Neoadjuvant treatment				.164[a]
Yes	12 (41.4%)	34 (28.1%)	46 (30.7%)	
No	17 (58.6%)	87 (71.9%)	104 (69.3%)	

Table. Continued

Characteristics	<45 (N = 29)	≥45 (N = 121)	Total (N = 150)	*P* value
Adjuvant chemotherapy				.771[a]
Yes	10 (34.5%)	38 (31.7%)	48 (32.2%)	
No	19 (65.5%)	82 (68.3%)	101 (67.8%)	
Missing	0	1	1	
Radiation				.025[a]
Yes	15 (51.7%)	36 (29.8%)	51 (34.0%)	
No	14 (48.3%)	85 (70.2%)	99 (66.0%)	
NGS tumor panel completed				.763[a]
Yes	10 (34.5%)	45 (37.5%)	55 (36.9%)	
No	19 (65.5%)	75 (62.5%)	94 (63.1%)	
Missing	0	1	1	
Family history of cancer				.273[a]
Yes	20 (69.0%)	70 (57.9%)	90 (60.0%)	
No/unknown	9 (31.0%)	51 (42.1%)	60 (40.0%)	
Family history of GI cancer				.580[a]
Yes	6 (20.7%)	31 (25.6%)	37 (24.7%)	
No	23 (79.3%)	90 (74.4%)	113 (75.3%)	
MMR				.275[a]
Positive	4 (13.8%)	9 (7.4%)	13 (8.7%)	
Negative	25 (86.2%)	112 (92.6%)	137 (91.3%)	
If MMR, IHC result				.118[a]
Normal	1 (25.0%)	0 (0.0%)	1 (7.7%)	
Abnormal	3 (75.0%)	9 (100.0%)	12 (92.3%)	
HRD				.751[a]
Positive	5 (17.2%)	18 (14.9%)	23 (15.3%)	
Negative	24 (82.8%)	103 (85.1%)	127 (84.7%)	
BRAF				.190[a]
Positive	1 (3.4%)	14 (11.6%)	15 (10.0%)	
Negative	28 (96.6%)	107 (88.4%)	135 (90.0%)	
Recurrence				.321[a]
Yes	0 (0.0%)	4 (3.3%)	4 (2.7%)	
No	29 (100.0%)	117 (96.7%)	146 (97.3%)	
Progression				.394[a]
Yes	4 (80.0%)	7 (58.3%)	11 (64.7%)	
No	1 (20.0%)	5 (41.7%)	6 (35.3%)	
Missing	24	109	133	
Deceased				.092[a]
Yes	0 (0.0%)	11 (9.1%)	11 (7.3%)	
No	29 (100.0%)	110 (90.9%)	139 (92.7%)	
Follow up (months)				.173[b]
Mean (SD)	14.8 (6.5)	21.5 (16.2)	20.2 (15.0)	
Median	14.5	15.1	15.0	
Range	4.5–29.9	1.6–87.8	1.6–87.8	

AJCC, American Joint Committee on Cancer; CEA, carcinoembryonic antigen; GI, gastrointestinal; IHC, immunohistochemistry; SD, standard deviation; VUS, variant of unknown significance.
[a]Pearson Chi-squared test.
[b]Kruskal-Wallis rank-sum test.

indicated that 12/150 (8.0%) tumors exhibited MSI-H. Most were MSS (n = 144, 96.0%) profile and had a TMB <10 muts/Mb (Supplementary Figures 2 and 3). MSI-H status was significantly associated with TMB-H ($P = 1.566e\text{-}09$). Additionally, assessment indicated an enrichment of MSI-H status in ESC patients ($P = .018$), patients with smoking history ($P = .012$), in tumors with mutated *BRAF* ($P = 2.837E\text{-}4$) and with at least 1 mutation in *BRAF/KRAS/NRAS* ($P = .013$). TMB-H was also enriched in ESC patients, particularly patients with stage II CRC (Supplementary Figure 2 and Supplementary Table 2).

Clinical Significance of Somatic Findings

Of 150 patients, 88 (58.7%) had potentially clinically significant variants detected in at least 1 of 5 categories across 52 genes (Figure 2). This included 95 variants with a Tier 1 therapeutic sensitivity or resistance implication in

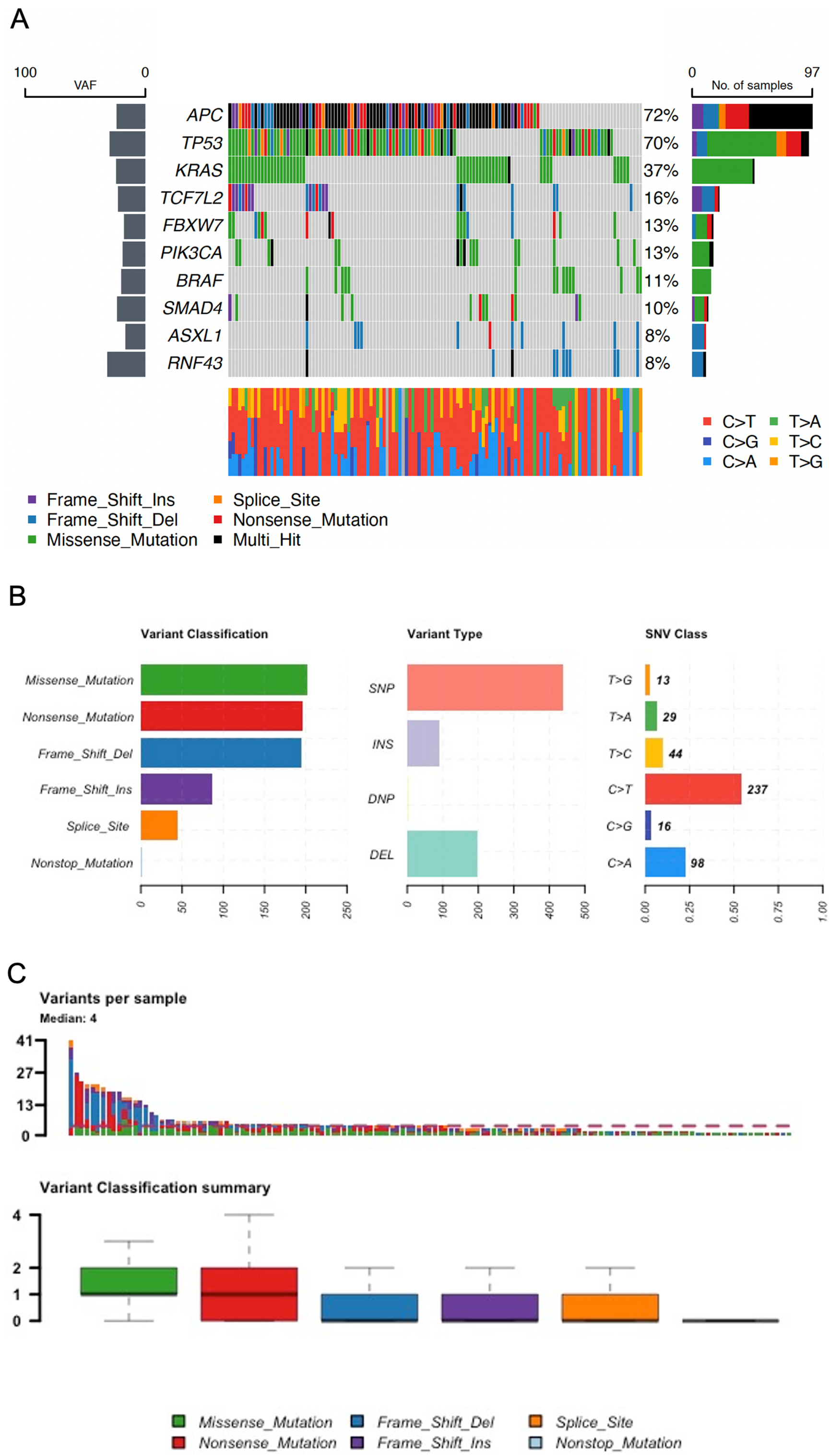

Figure 1. Summary of somatic findings in patients with positive findings. For patients with positive somatic findings (n = 132), (A) details the most frequently altered genes in the cohort, including the mean variant allele frequency (VAF) and the proportion of each variant type identified, as well as the proportion of transitions and transversions seen in each patient. Each column in the graph represents a single patient. In (B), variant types and functional classification of variants across the cohort are summarized. In (C), the per sample distribution of variant count and type is shown.

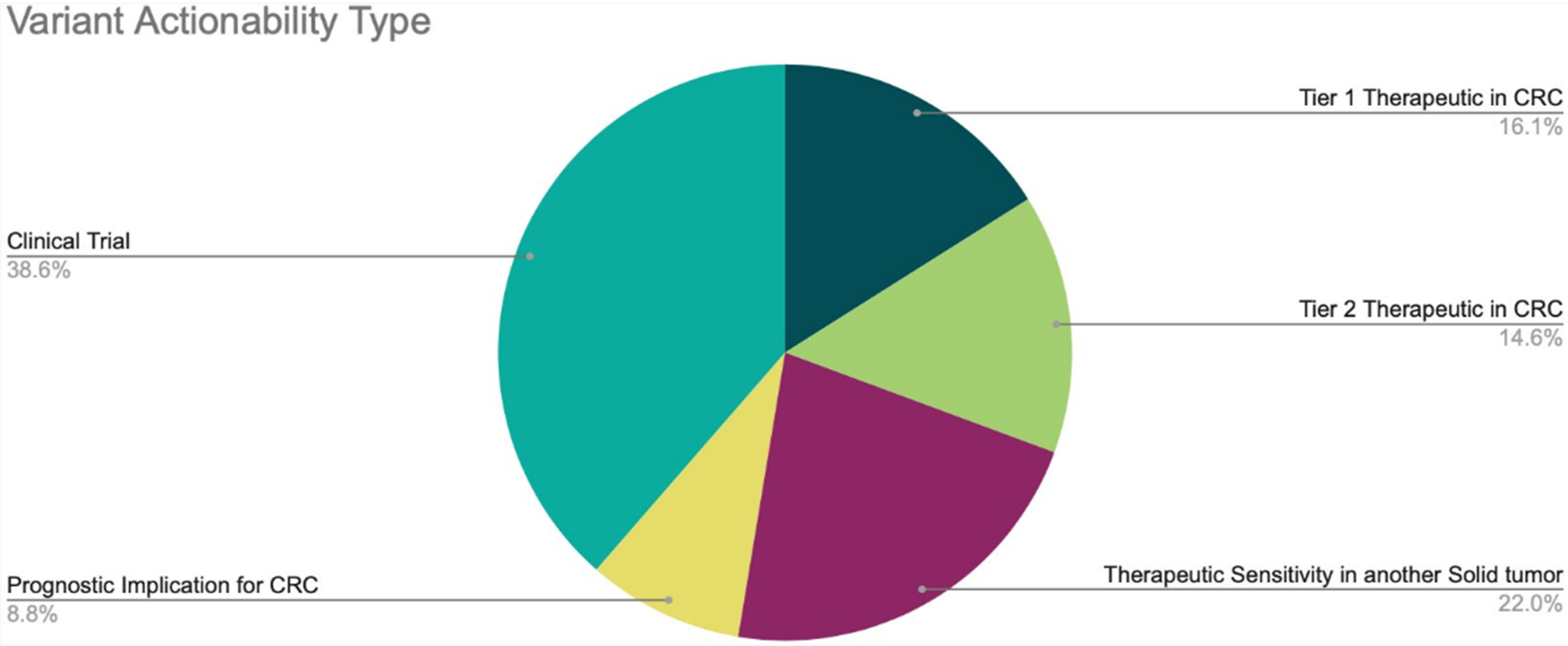

Figure 2. Clinically significant types of somatic variants. In the figure, variants are classified accordingly with actionability, clinical trials available, and prognostic implications.

CRC, 86 variants with a Tier 2 therapeutic sensitivity or resistance implication in CRC, 130 variants with a therapeutic sensitivity in another solid tumor type, 52 variants with a prognostic implication in CRC, and 228 with clinical trial associations for CRC or a solid tumor (591 total associations across 289 variants). Of 289 variants detected across 52 genes, the most frequently detected were variants in *KRAS* (51), *PIK3CA* (21), *FBXW7* (18), *BRAF* (15), *PTEN* (15), and *RNF43* (15). Twenty-seven of the 88 cases with somatic findings were TMB-H with 12 (44.4%) of those patients also characterized as MSI-H. Both TMB-H and MSI-H status are eligible for immune checkpoint inhibitor therapy and confer eligibility for additional clinical trials. For 5/88 cases, TMB-H status was the only clinically significant finding.

Combined Germline and Somatic

Ten percent (15/150) of patients were negative for both germline and somatic findings of established or potential clinical significance. In total 76.0% (114/150) had either a GGT (n = 3) or CGP (n = 111) clinically significant findings, while 14.0% (21/150) cases had both CGP and GGT clinically significant findings. Among EOC patients (n = 29), 6 (20.7%) had PGVs, in which all patients had additional Tier 1 or Tier 2 variants, while 20 (70.0%) only had somatic Tier 1 or Tier 2 variants, and 3 (10.3%) were negative for PGVs or somatic variants of clinical significance. Among ESC patients (n = 105), 18 (17.1%) had PGVs in which 17/18 (94.4%) had additional somatic Tier 1 or Tier 2 variants, 76 (72.4%) only had somatic Tier 1 or Tier 2 variants, and 11 (10.5%) had both (Figure 3). Combined GGT and CGP testing significantly increased clinically significant findings over GGT alone (74.0%), but only modestly over somatic testing alone (2.7%) (Figure 4). These findings were similar when subsetting for ESC (increased utility over GGT alone: 72.4%, increased utility over somatic testing alone: 2.9%) and EOC increased utility over GGT alone: 69.0%, increased utility over somatic testing alone: 6.9%).

Pathway Analysis

Pathway analysis of CGP findings indicated an enrichment of mutations in DNA repair pathways ($P = 6.420$ E^{-10}; FDR = 1.520 E^{-07}) and the cell cycle ($P = 1.230$ E^{-07}; FDR:1.330 E^{-05}). Alterations were frequent in the double strand break response ($P = 1.650$ E^{-06}; FDR: 1.520 E^{-04}); largely focused on the homologous recombination repair (HRR) pathway (P = 3.770 E^{-14}–1.530 E^{-08}; FDR: 1.570 E^{-11}–2.540 E^{-06}). Alterations associated with the *TP53* DNA damage pathway were also significantly enriched ($P = 1.110$ E^{-16}; FDR: 1.850 E^{-13}). Additionally, oncogenic signaling in the rat sarcoma virus/rapidly accelerated fibrosarcoma (P = 1.710 E^{-9}–2.770 E^{-6}; FDR: 3.140 E^{-7}–2.410 E^{-4}) and PI3K pathways (P = 1.710 E^{-9}–3.990 E^{-6}; FDR: 3.140 E^{-7}–3.050 E^{-4}) were frequently altered in the cohort. No difference was observed in pathway enrichment between early and late disease onset (Supplementary Tables 3–5; Figure 5).

Mutations in 20 genes were found to significantly co-occur with another gene within the dataset; no statistically significant mutually exclusive relationships were identified. Previously unreported co-occurrence relationships identified in the cohort included *ATM-B2M, MSH6-B2M, RNF43-CDK12* (P=<.001) (Supplementary Table 3).

Mutational Signature Analysis

Mutational signature analysis identified 3 main functional mutational signatures (*POLE*, defective MMR [dMMR], and defective homologous recombination deficiency [HRD]) (Supplementary Table 4). This analysis highlighted the driving effect of mutations within the HRR, MMR and POLE pathways. Two patients with EOC exhibited *POLE* mutation signatures (SBS 10a and 10b) and had *POLE* hotspot mutations. Sequencing identified a somatic *POLE* c.857C>G p. Pro286Arg mutation in both patients at allele

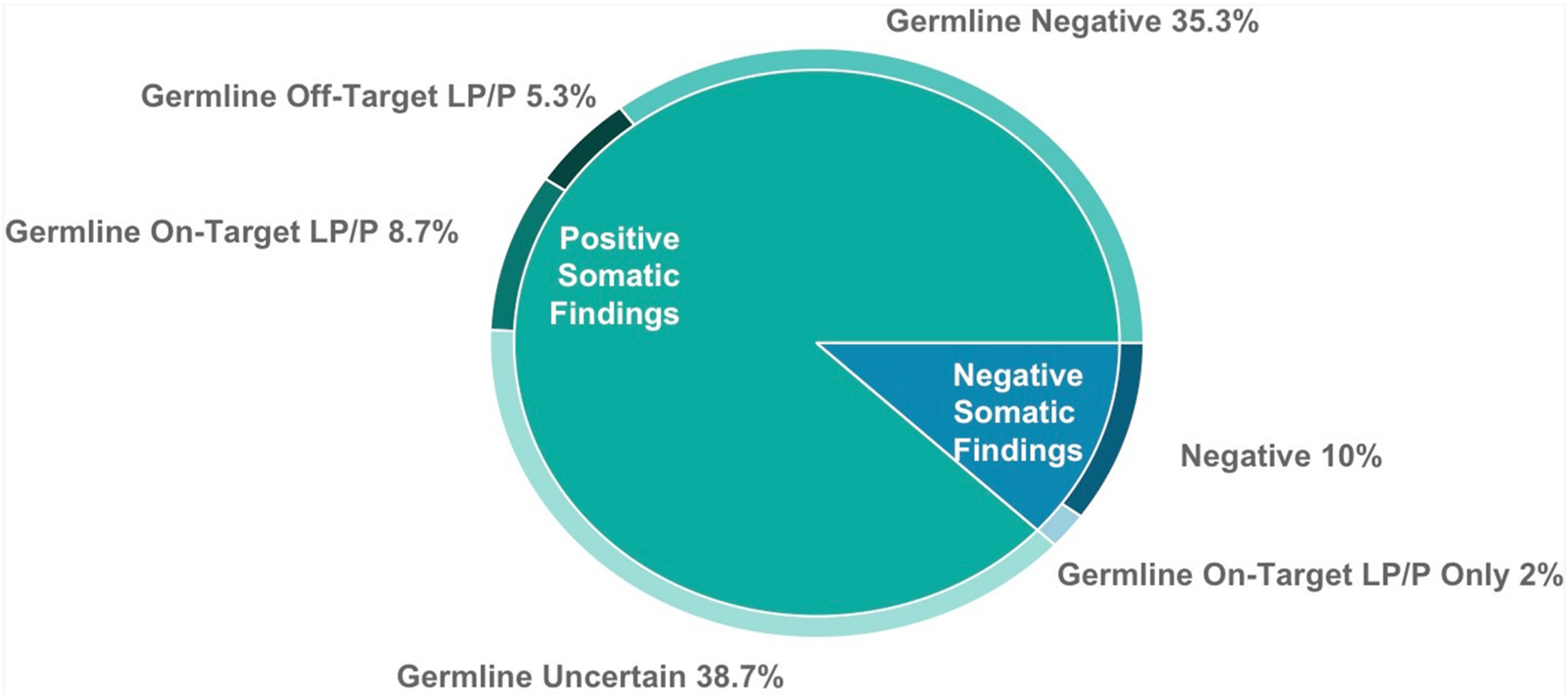

Figure 3. Proportion of germline and somatic findings in cohort. Germline pathogenic/likely pathogenic (LP/P) findings are further detailed as on or off target for colorectal cancer.

frequencies of 24.5% and 25.1%, respectively. One patient was diagnosed with stage II colon cancer at age 44 years and had a TMB value of 245.9 muts/Mb, indicating hypermutator status. Another patient was diagnosed with stage III rectal cancer at age 39 years. The patient had a family history of cancer but not gastrointestinal cancer. While the sample failed TMB QC and therefore TMB was not reported, the patient did exhibit a high number of variants post-filtering for variant review indicating that the patient may have had a high TMB.

A 65-year-old rectal cancer patient exhibited a strong HRD mutational signature (SBS 3). The results of CGP

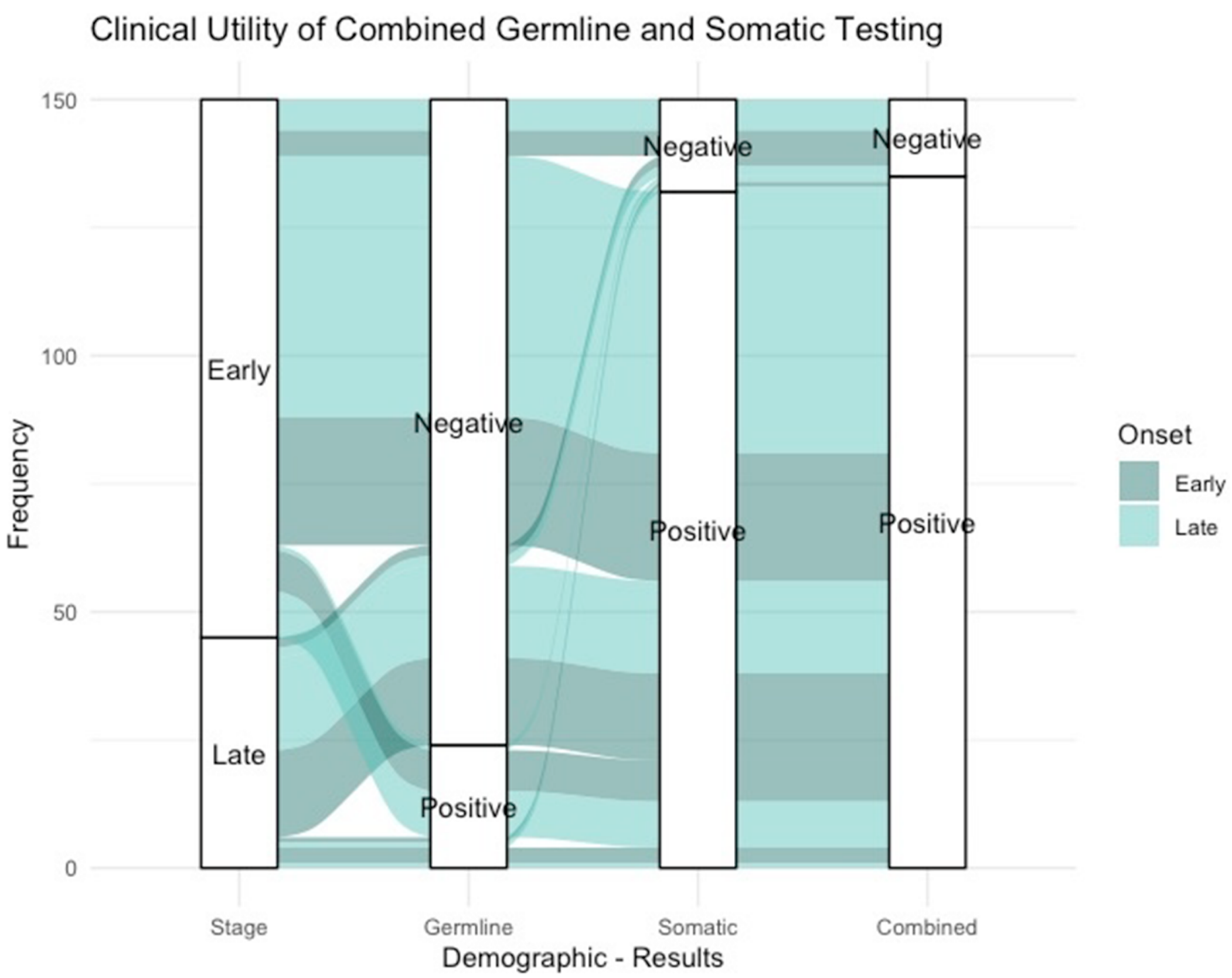

Figure 4. Clinical utility of combined germline and somatic testing. Frequency of patients with somatic and germline testing, broken down by age of onset and stage of patients. The increased utility of the CGP-GGT testing is indicated as well. Early-stage considered localized disease (stages 0-3).

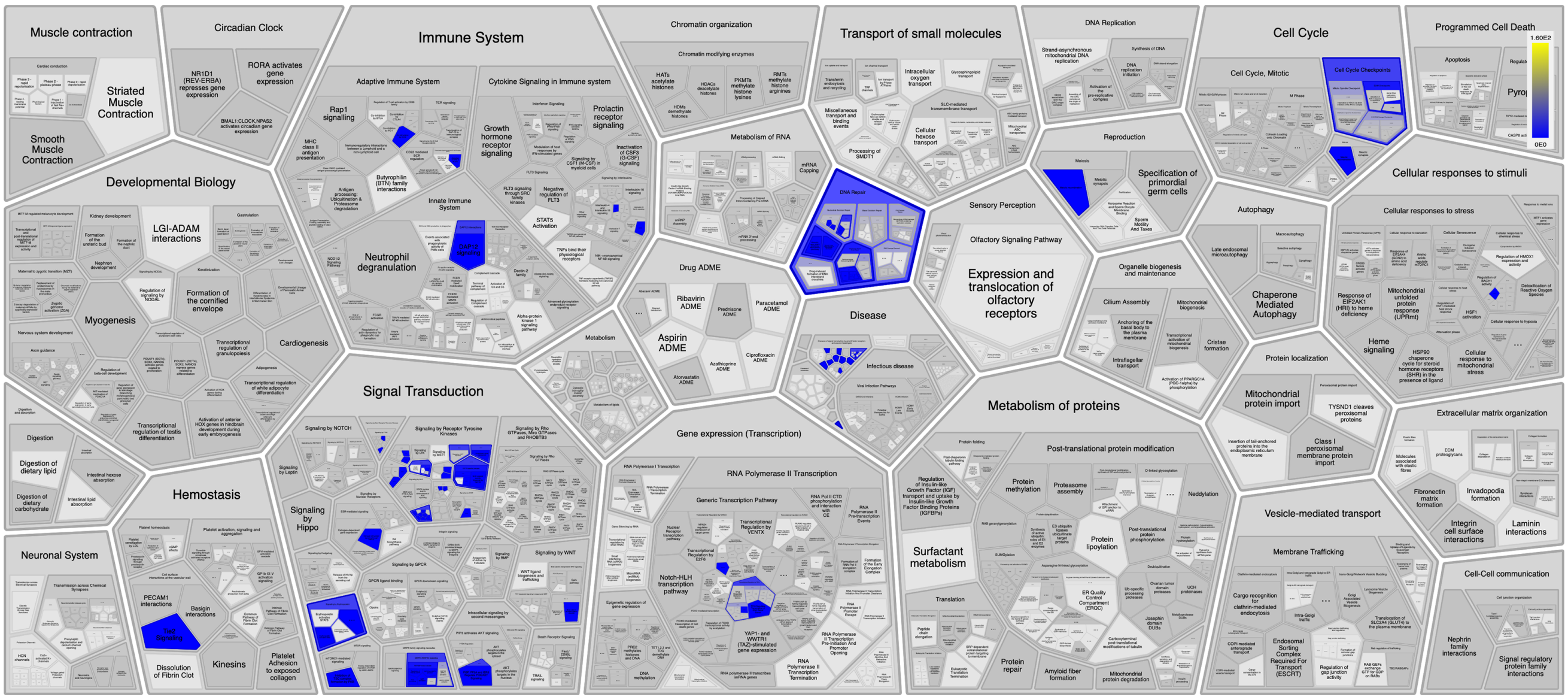

Figure 5. Pathway enrichment analysis of cohort. Figure highlights the pathways enriched in mutations in the cohort, with analysis highlighting an abundance of genes within the cell cycle and DNA damage pathways being altered in the dataset.

indicated that the tumor was MSS and had a high TMB of 15.7; however, no HRD pathway mutations were detected.

Additionally, 12 patients were found to exhibit dMMR mutational signatures. Of these, 4 (33.3%) patients had oncogenic dMMR mutations identified. Three of the 4 had both single-base substitutions (SBS) signature 6 and 26 identified, which are associated with dMMR and MSI. Additionally, all had high TMB rates and were MSI-H. A patient diagnosed with stage III rectal cancer at age 27 years had a heterozygous germline *MSH2* c.187del (p. Gly62_Val63insTer) pathogenic variant, while the other 3 patients had somatic variants in dMMR genes. Overall, the data indicate that these 4 tumors were driven by dMMR mutations. Eight tumors with dMMR mutational signatures (SBS signature 26, with another case also exhibiting signature 6) did not have GGT or CGP MMR gene variants identified. These 8 samples had low TMB and were MSS. These patients were all diagnosed at ≤ 62 years old, including 2 EOC cases that were negative for any PGVs. Additionally, 11 samples were positive for MMR variants, but the dMMR mutational signature was not detected. In these 11 cases, TMB was high in 63.6% (n = 7) cases.

Disease-Specific Outcomes

Comparing median overall survival, neither any statistical difference was identified between patients with or without PGVs ($P = .630$) nor in patients with or without somatic variants ($P = .600$) (Supplementary Figures 4 and 5). For patients with resectable disease, recurrence-free survival was also not associated with germline or somatic alterations ($P = .110$ and $P = .490$), respectively (Supplementary Figures 6 and 7).

Discussion

The combination of exome-based CGP with comprehensive GGT realized in this study, in a prospective multicenter cohort of patients, provided valuable insights into the impact of paired genomic profiling in unselected patients with CRC. Furthermore, with the integration of clinical demographics, we identified associations with molecular characteristics including TMB-H with ESC. Combining GGT and CGP increased the likelihood of identifying clinically significant findings. Finally, exome CGP analysis identified enrichment of mutations in DNA repair and cell cycle pathways in the overall cohort.

In this cohort of unselected patients with CRC, 16.0% of the patients harbored PGVs. The incidence of germline findings was slightly higher in patients less than 45 years old compared to patients older than 45 years but did not reach statistical significance (20.7% vs 14.9%, $P = .443$), which is consistent with other studies.[28] CRC in EOC tended to be more frequently left sided (75.9% vs 69.4%), particularly rectal cancers (55.2% vs 40.5%), however, without reaching statistical significance ($P = .354$). Somatic findings were less likely to be identified in EOC, consistent with other studies, suggesting that different treatment strategies based only on age in CRC are not justified due to any clear genomic differences when compared to sporadic CRC with average age of onset.[29]

Active smoking was statistically related to MSI-H and *BRAF* mutations. This is consistent with data on lung cancer, where *BRAF* mutations are related to smoking status.[30] Correlations with MSI-H and *BRAF* mutations are well known, and studies combining immunotherapy with *BRAF* inhibitors, in patients with metastatic CRC, are underway.[31]

MSI-H and TMB-H were statistically significant associated with ESC. This information today is very important considering that immunotherapy is being evaluated in the adjuvant setting in trials like ATOMIC.[32]

The utility of paired GGT and CGP testing was high (≈70%). Among EOC patients, 70.0% had only somatic Tier 1 or Tier 2 variants, and among ESC patients, 72.4% had only somatic Tier 1 or Tier 2 variants. Overall, the impact of somatic testing in paired GGT and CGP was higher than GGT alone, in terms of detecting actionability. However, GGT alone increased utility in detection of genomic alterations in 2.9% in ESC and 6.9% in EOC, warranting further investigation in larger cohorts with different populations to understand broadly the impact of those findings in early cancer detection and CRC prevention.

The median overall survival and recurrence-free survival was not associated with germline or somatic findings; however, these findings should be taken with caution considering that precision medicine in CRC is in constant evolution. Since these data were collected, BRAF inhibitors, immunotherapy, and KRAS inhibitors were incorporated into the treatment landscape of advanced CRC.[33–35] Approximately, only 10% of patients were negative for variants of established or potential clinical significance.

The pathway and mutational signature analysis revealed key findings, highlighting frequent somatic mutations in DNA repair and cell cycle pathways. Notably, the double strand break response (especially HRR) and TP53 DNA damage pathways showed significant enrichment of alterations. Oncogenic signaling in rat sarcoma virus/rapidly accelerated fibrosarcoma and PI3K pathways was also commonly altered. However, no pathway enrichment differences were observed between early and late disease onset. Co-occurrence of mutations in 20 genes was identified, including novel relationships like *ATM-B2M*, *MSH6-B2M*, and *RNF43-CDK12*, while no mutually exclusive mutations were found. Mutational signature analysis revealed 3 primary signatures implicating alterations in these pathways as driving mutations in CRC: POLE, dMMR, and HRD. These pathways are known as key drivers of CRC when altered and have emerging or established implications for prognosis and therapy. Oncogenic *POLE* mutations have been shown in multiple studies to be associated with clinical benefit to immunotherapy.[36–38] Patients with alterations in the HHR pathway have been shown to exhibit longer overall survival in CRC.[39] Nivolumab alone or in combination with ipilimumab has been approved by the FDA for patients with dMMR metastatic CRC that have progressed or is unresectable. Identifying alterations or mutational signatures in these pathways can aid in patient management decisions regarding prognosis and therapy.

Mutational signature analysis additionally detected mutational signature SBS26 in 8 patients without dMMR variant alterations, as well as 1 patient with SBS3 in a patient without mutations in the HRD pathway identified by sequencing. SBS26 is 1 of seven mutational signatures associated with dMMR and MSI, while Signature SBS3 is strongly associated with germline and somatic *BRCA1* and *BRCA2* mutations as well as *BRCA1* promoter methylation in breast, pancreatic, and ovarian cancers. Potentially, these patients may be affected by a genomic alteration out of scope of this assay, such as epigenetic silencing by promoter methylation or may have had loss of heterozygosity of an HRD or MMR gene. These findings highlight the importance of mutational signature analysis which could help to identify additional patients eligible for targeted treatments. Additionally, some patients showed MMR variants without the dMMR mutational signature, with high TMB in 63.6% of those cases, indicating that these patients may benefit from immunotherapy. Furthermore, the lack of dMMR mutational signature in this subset of patients does indicate that the mutational signature analysis may be prone to false negatives.

As for some limitations of the study, the cohort composition, the overall patients' characteristics seen at the multiple Mayo Clinic sites that participated in this study may not mirror those in all regions of the country limiting generalizability. Sample size is relatively small, furthermore, lack of long-term follow-up to address the real impact in the cancer-mortality and morbidity associated with systemic treatments, personalized interventions or surgeries related to the findings.

This research has also strengths. This is a multicenter, prospective, universal paired genetic testing study. Recruitment occurred regardless of stage or family history of cancer. This study represents one of the most comprehensive universal genomic analyses performed in a prospective multicentered cohort of unselected patients with CRC and suggests the power of the combination of variant assessment and mutational signatures analysis for identifying the full spectrum of patients that may benefit from therapies targeting the dMMR and HRD pathways and for immunotherapy.

Conclusion

Combined GGT-CGP increases clinical utility for patients with CRC, and these data suggest combined GGT-CGP should be considered for all patients with CRC and may be considered for broader patient populations. For patients with EOC experiencing poorer outcomes, there is a higher likelihood of actionability with paired testing. CGP-GGT also drives germline resolution for tumor mutations in hereditary cancer genes, expediting clinical recommendations.

Supplementary Materials

Material associated with this article can be found, in the online version, at https://doi.org/10.1016/j.gastha.2026.101093.

References

1. Bray F, Laversanne M, Sung H, et al. Global cancer statistics 2022: GLOBOCAN estimates of incidence and

mortality worldwide for 36 cancers in 185 countries. CA Cancer J Clin 2024;74(3):229–263.
2. Dizon DS, Kamal AH. Cancer statistics 2024: all hands on deck. CA Cancer J Clin 2024;74:1.
3. Patel SG, Karlitz JJ, Yen T, et al. The rising tide of early-onset colorectal cancer: a comprehensive review of epidemiology, clinical features, biology, risk factors, prevention, and early detection. Lancet Gastroenterol Hepatol 2022;7(3):262–274.
4. Uson PLS Jr, Riegert-Johnsonet D, Boardman L, et al. Germline cancer susceptibility gene testing in unselected patients with colorectal adenocarcinoma: a multicenter prospective study. Clin Gastroenterol Hepatol 2022;20(3):e508–e528.
5. Hodan R, Rodgers-Fouche L, Arora S, et al. Patterns of germline and somatic testing after universal tumor screening for Lynch syndrome: a clinical practice survey of active members of the Collaborative Group of the Americas on Inherited Gastrointestinal Cancer. J Genet Couns 2022;31(4):949–955.
6. Muller C, Lee SM, Barge W, et al. Low referral rate for genetic testing in racially and ethnically diverse patients despite universal colorectal cancer screening. Clin Gastroenterol Hepatol 2018;16(12):1911–1918.
7. Samadder NJ, Riegert-Johnson D, Boardmanet L, et al. Comparison of universal genetic testing vs guideline-directed targeted testing for patients with hereditary cancer syndrome. JAMA Oncol 2021;7(2):230–237.
8. Lincoln SE, Hambuch T, Zook JM, et al. One in seven pathogenic variants can be challenging to detect by NGS: an analysis of 450,000 patients with implications for clinical sensitivity and genetic test implementation. Genet Med 2021;23(9):1673–1680.
9. Lincoln SE, Truty R, Lin C-F, et al. A rigorous interlaboratory examination of the need to confirm next-generation sequencing–detected variants with an orthogonal method in clinical genetic testing. J Mol Diagn 2019;21(2):318–329.
10. Mc Kenna A, Hanna M, Banks E, et al. The Genome analysis toolkit: a MapReduce framework for analyzing next-generation DNA sequencing data. Genome Res 2010;20(9):1297–1303.
11. Garrison E, Marth G. Haplotype-based variant detection from short-read sequencing. arXiv 2012:Preprint arXiv:1207.3907 [q-bio.GN].
12. Jacobs KB, Paul JS, Nilsen GB, et al. In: Presented at the cold Spring Harbor biology of genomes meeting. CNVitae—accurate detection of small and large copy number events from DNA capture sequence data. New York: Cold Spring Harbor, 2013. Abstract 157. Available at: https://expydoc.com/doc/4519833/accurate-detection-of-small-and-large-copy-number. Accessed August 27, 2026.
13. Nord AS, Lee M, King M-C, et al. Accurate and exact CNV identification from targeted high-throughput sequence data. BMC Genomics 2011;12:1–10.
14. Nykamp K, Anderson M, Powers M, et al. Sherloc: a comprehensive refinement of the ACMG–AMP variant classification criteria. Genet Med 2017;19(10):1105–1117.
15. Chen S, Zhou Y, Chen Y, et al. Fastp: an ultra-fast all-in-one FASTQ preprocessor. Bioinformatics 2018;34(17):i884–i890.
16. Li H, Durbin R. Fast and accurate short read alignment with burrows–wheeler transform. Bioinformatics 2009;25(14):1754–1760.
17. Mose LE, Perou CM, Parker JS. Improved indel detection in DNA and RNA via realignment with ABRA2. Bioinformatics 2019;35(17):2966–2973.
18. Lai Z, Markovetz A, Ahdesmaki M, et al. VarDict: a novel and versatile variant caller for next-generation sequencing in cancer research. Nucleic Acids Res 2016;44(11):e108.
19. Van der Auwera GA, O'Connor BD. In: Genomics in the cloud: using docker, GATK, and WDL in terra. 1st ed. O'Reilly Media, 2020.
20. Stromberg M, Roy R, Lajugie J, et al. Nirvana: clinical grade variant annotator. In: Proceedings of the 8th ACM International conference on Bioinformatics, Computational Biology, and Health Informatics (ACM-BCB '17); 2017:596, Association for Computing Machinery, New York, NY, USA.
21. Li MM, Datto M, Duncavage E, et al. Standards and guidelines for the interpretation and reporting of sequence variants in cancer: a joint consensus recommendation of the Association for Molecular Pathology. American Society of Clinical Oncology, and College of American Pathologists. J Mol Diagn 2017;19(1):4–23.
22. Riester M, Singh AP, Brannon AR, et al. PureCN: copy number calling and SNV classification using targeted short read sequencing. Source Code Biol Med 2016;11:1–13.
23. Sun JX, He Y, Sanford E, et al. A computational approach to distinguish somatic vs. germline origin of genomic alterations from deep sequencing of cancer specimens without a matched normal. PLoS Computat Biol 2018;14(2):e1005965.
24. Ripley BD. The R project in statistical computing. MSOR Connect Newsletter LTSN Maths, Stats OR Netw 2001;1(1):23–25.
25. Mayakonda A, Lin D-E, Assenov Y, et al. Maftools: efficient and comprehensive analysis of somatic variants in cancer. Genome Res 2018;28(11):1747–1756.
26. R Core Team. In: R: a language and environment for statistical computing. Vienna: R. Foundation for Statistical Computing, 2013. http://www.R-project.org/. Accessed August 27, 2026.
27. Griss J, Viteri G, Sidiropoulos K, et al. ReactomeGSA-efficient multi-omics comparative pathway analysis. Mol Cell Proteomics 2020;19(12):2115–2125.
28. Fantini D, Vidimar V, Yu Y, et al. MutSignatures: an R package for extraction and analysis of cancer mutational signatures. Sci Rep 2020;10(1):18217.
29. Cercek A, Chatila WK, Yaeger R, et al. A comprehensive comparison of early-onset and average-onset colorectal cancers. JNCI J Natl Cancer Inst 2021;113(12):1683–1692.
30. Sasaki H, Shitara M, Yokota K, et al. Braf and erbB2 mutations correlate with smoking status in lung cancer patients. Exp Ther Med 2012;3(5):771–775.
31. Elez E, Kopetz S, Tabernero J, et al. SEAMARK: phase II study of first-line encorafenib and cetuximab plus pembrolizumab for MSI-H/dMMR BRAF V600E-mutant mCRC. Future Oncol 2024;20(11):653–663.

32. Sinicrope FA, Ou F-S, Arnold D, et al. Randomized trial of standard chemotherapy alone or combined with atezolizumab as adjuvant therapy for patients with stage III deficient DNA mismatch repair (dMMR) colon cancer (Alliance A021502; ATOMIC). J Clin Oncol 2025;43:LBA1.
33. André T, Elez E, Henz H-J, et al. Nivolumab plus ipilimumab versus nivolumab in microsatellite instability-high metastatic colorectal cancer (CheckMate 8HW): a randomised, open-label, phase 3 trial. Lancet 2025; 405(10476):383–395.
34. Siena S, Yamaguchi K, Ruffinelli Rodriguez JC, et al. 505O Sotorasib (soto), panitumumab (pani) and FOLFIRI in the first-line (1L) setting for KRAS G12C–mutated metastatic colorectal cancer (mCRC): safety and efficacy analysis from the phase Ib CodeBreaK 101 study. Ann Oncol 2024;35(Suppl 2):S429–S430.
35. Elez E, Yoshino T, Shen L, et al. Encorafenib, cetuximab, and mFOLFOX6 in BRAF-mutated colorectal cancer. N Engl J Med 2025;392(24):2425–2437.
36. Garmezy B, Gheeya J, Lin HY, et al. Clinical and molecular characterization of POLE mutations as predictive biomarkers of response to immune checkpoint inhibitors in advanced cancers. JCO Precis Oncol 2022;6: e2100267.
37. Wang F, Zhao Q, Yang YN, et al. Evaluation of POLE and POLD1 mutations as biomarkers for immunotherapy outcomes across multiple cancer types. JAMA Oncol 2019;5(10):1504–1506.
38. Ambrosini M, Rousseau B, Manca P, et al. Immune checkpoint inhibitors for POLE or POLD1 proofreading-deficient metastatic colorectal cancer. Ann Oncol 2024; 35(7):643–655.
39. Moretto R, Elliott A, Zhang J, et al. Homologous recombination deficiency alterations in colorectal cancer: clinical, molecular, and prognostic implications. JNCI J Natl Cancer Inst 2022;114(2):271–279.

Received March 9, 2026. Accepted August 6, 2026.

Correspondence:
Address correspondence to: Jewel Samadder, MD, MSc, Division of Gastroenterology and Hepatology, Department of Medicine, Mayo Clinic, 5881 E Mayo Blvd, Phoenix, Arizona 85054. e-mail: samadder.jewel@mayo.edu.

Authors' Contributions:
Pedro Luiz Serrano Uson Junior, Jacquelyn Reuther, and Jianhua Zhao: Writing, conceptualization, and data curation. Michael A. Golafshar, Megan H. Hawley, Katie L. Kunze, Emily Lavy, and Sarah M. Young: Editing, review, methodology, and investigation. Tanios Bekaii-Saab, Christina Wu, W. Michael Korn, Edward D. Esplin, Robert Daber, and Jewel Samadder: Project administration, resources, and reviewing. All authors accepted the final form of the manuscript.

Conflicts of Interest:
The authors disclose no conflicts.

Funding:
This study was supported by Mayo Practice Transformation Grant, Mayo Clinic Center for Individualized Medicine, Desert Mountain Members'CARE Foundation, David and Twila Woods Foundation, and Faculty Career Development Award from the Gerstner Foundation (Jewel Samadder).

Ethical Statement:
This study was approved by the Mayo Clinic Institutional Review Board (19-010300). All patients provided written informed consent. Data were deidentified except to investigators of the study.

Data Transparency Statement:
Data and study materials are not directly available. Analytic methods are available upon request.

Reporting Guidelines:
Reporting Guidelines were not applicable for this article type.

Gastro Hep Advances, Volume 5

Supplemental information

Paired Exome-Based Comprehensive Genomic Profiling and Germline Genetic Testing for Unselected Patients With Colorectal Cancer in a Multicenter Prospective Study

Pedro Luiz Serrano Uson, Jacquelyn Reuther, Michael A. Golafshar, Megan H. Hawley, Emily Lavy, Sarah M. Young, Tanios Bekaii-Saab, Christina Wu, W. Michael Korn, Edward D. Esplin, Jianhua Zhao, Katie L. Kunze, Robert Daber, and Jewel Samadder

Supplementary Tables and Figures:

Supplementary Table 1. Germline Actionable Results

Sample ID	Age	AJCC 8th Staging	METS	Positive Findings	Clinical Trials	Precision Therapy in another tumor type	Management Guidelines
Patient 1	68	3	No	ATM Exon 62-63 deletion	Y	Y	Y
Patient 2	48	4	Yes	BRCA2 p.Glu1493fs	Y	Y	Y
Patient 3	68	2	No	BRIP1 c.2097+1G>C	N	N	Y
Patient 4	40	4	Yes	CDC73 Exon 15 deletion	N	N	Y
Patient 5	57	3	No	CHEK2 Exon 3-4 duplication	Y	Y	Y
Patient 6	49	2	No	MSH2* c.942+3A>T	Y	N	Y
Patient 7	44	2	No	MSH2* Exon 1-6 deletion	Y	N	Y
Patient 8	46	1	No	MSH2* p.Ser554Asn	Y	N	Y
Patient 9	27	3	No	MSH2* p.Val63Ter	Y	N	Y
Patient 10	27	2	No	MSH6* c.3556+1G>A	Y	N	Y
Patient 11	73	4	Yes	RAD51D p.Asp256ThrfsTer54	Y	Y	Y
Patient 12	46	4	Yes	SDHA p.Arg361His	N	N	Y
Patient 13	75	2	No	SDHD p.Pro81Leu	N	N	Y
Patient 14	47	3	No	TP53 p.Gly245Ser	N	N	Y

*Indicative of possible dMMR so IHC should be performed to determine eligibility for ICIs.

Supplementary Table 2. Statistical Associations Regarding Molecular Biomarkers and Clinicopathologic characteristics.

Legend: Table describes the Fisher's Exact Test (*P-value*) results for various statistical associations between molecular biomarkers and patient characteristics.

	MSI	TMB	Onset	KRAS	BRAF	RAF/RAF	HRD
Stage	*.018*	*.007*	*.089*	*.249*	*.552*	*.060*	***.050***
Age of Onset	*.358*	*.822*	-	*.856*	*.403*	*.384*	*.646*
Gender	*.762*	*.500*	*1.000*	*.471*	*.407*	*.162*	*.498*
Hypertension (HT)	*.759*	*.816*	*5.770 E-05*	*.712*	*.256*	*1.000*	*.817*
Smoking	*.012*	*.173*	*.012*	*.257*	***.027***	*.860*	***.033***
Obesity	*.534*	*.356*	*.466*	*.459*	*.573*	*1.000*	*1.000*
Diabetes	*.599*	*.378*	*.003*	*.212*	*1.000*	*.214*	*1.000*
Family history of cancer	*.069*	*.377*	*.078*	*.369*	*.158*	*1.000*	*1.000*
Family history of GI cancer	*.166*	*1.000*	*1.000*	*.194*	*.528*	*.433*	*.114*
TMB	*1.570 E-9*	-	*See TMB*	*.823*	*.009*	*.044*	*.001*
MSI	-	*See MSI*	*See MSI*	*1.000*	*2.840 E-4*	*.013*	*1.30E-6*

Supplementary Table 3. Co-Occurrence Relationships Identified in Cohort

Legend: Table identifies the statistically significant co-occurrence relationships identified in the cohort.

Gene 1	Gene 2	Samples WT for both genes	Gene 1 mutant samples	Gene 2 mutant samples	Gene 1 and 2 mutant samples	Log2 Odds Ratio	*P-Value*	Q-Value
RNF43	ARID1A	130	4	3	7	>3	*<.001*	*<.001*
ATM	B2M	136	2	2	4	>3	*<.001*	*.010*
MSH6	B2M	132	6	1	5	>3	*<.001*	*.010*
ARID1A	BCOR	134	6	0	4	>3	*<.001*	*.010*
RNF43	CDK12	133	7	0	4	>3	*<.001*	*.010*
ARID1A	ERCC5	134	6	0	4	>3	*<.001*	*.010*
BRCA2	FAT1	136	2	2	4	>3	*<.001*	*.010*
RNF43	MSH6	128	5	5	6	>3	*<.001*	*.010*
BRAF	RNF43	125	8	4	7	>3	*<.001*	*.010*
MSH6	TGFBR2	133	7	0	4	>3	*<.001*	*.010*
RNF43	TGFBR2	133	7	0	4	>3	*<.001*	*.010*
LRP1B	FAT1	135	3	2	4	>3	*<.001*	*.014*
KMT2A	ERCC5	139	1	1	3	>3	*<.001*	*.015*
PBRM1	FAT1	138	0	3	3	>3	*<.001*	*.017*
ARID1A	PTCH1	133	6	1	4	>3	*<.001*	*.023*
BCOR	CREBB	138	1	2	3	>3	*<.001*	*.026*

PTCH1	ERCC5	138	2	1	3	>3	*<.001*	*.026*
PBRM1	LRP1B	137	0	4	3	>3	*<.001*	*.026*
RNF43	PTCH1	132	7	1	4	>3	*<.001*	*.028*
FAT1	RB1	137	3	1	3	>3	*<.001*	*.047*
PTEN	TET1	135	6	0	3	>3	*<.001*	*.047*

Supplementary Table 4: Pathways identified

Pathway name	Entities found	Entities total	Interactors found	Interactors total	Entities ratio	Entities *P-value*	Entities FDR	Reactions found	Reactions total	Reactions ratio
TP53 Regulates Transcription of DNA Repair Genes	25	86	11	150	.006	1.110 E-16	1.850 E-13	17	17	.001
Defective homologous recombination repair (HRR) due to PALB2 loss of function	11	25	0	5	.002	3.770 E-14	1.570 E-11	2	2	1.310 E-4
Defective HDR through Homologous Recombination Repair (HRR) due to PALB2 loss of BRCA1 binding function	11	25	0	5	.002	3.770 E-14	1.570 E-11	1	1	6.550 E-5
Defective HDR through Homologous Recombination Repair (HRR) due to PALB2 loss of BRCA2/RAD51/RAD51C binding function	11	25	0	5	.002	3.770 E-14	1.570 E-11	1	1	6.550 E-5
Impaired BRCA2 binding to PALB2	11	24	3	17	.002	3.560 E-13	1.180 E-10	1	1	6.55 E-5
Defective homologous recombination repair (HRR) due to BRCA1 loss of function	11	25	3	17	.002	4.730 E-13	1.310 E-10	1	1	6.550 E-5
Diseases of DNA repair	18	54	19	228	.003	6.420 E-10	1.520 E-7	17	34	.002
Signaling by PDGFRA transmembrane, juxtamembrane and kinase domain mutants	7	19	0	0	.001	1.700 E-9	3.140 E-7	7	7	4.590 E-4

Signaling by PDGFRA extracellular domain mutants	7	19	0	0	.001	1.700 E-9	3.140 E-7	7	7	4.590 E-4
Defective homologous recombination repair (HRR) due to BRCA2 loss of function	11	41	5	67	.003	1.530 E-8	2.540 E-6	4	4	2.620 E-4
Impaired BRCA2 binding to RAD51	9	35	4	28	.002	1.840 E-8	2.780 E-6	1	1	6.550 E-5
RAS signaling downstream of NF1 loss-of-function variants	5	9	0	0	5.710 E-4	5.100 E-8	7.030 E-6	1	1	6.550 E-5
GRB2 events in ERBB2 signaling	6	21	0	0	.001	1.120 E-7	1.330 E-5	4	4	2.620 E-4
G2/M DNA damage checkpoint	11	80	6	60	.005	1.230 E-7	1.330 E-5	11	12	7.860 E-4
Signaling by KIT in disease	7	28	1	9	.002	1.290 E-7	1.330 E-5	26	26	.002
Signaling by phosphorylated juxtamembrane, extracellular and kinase domain KIT mutants	7	28	1	9	.002	1.290 E-7	1.330 E-5	11	11	7.210 E-4
Signaling by PDGFR in disease	7	27	1	10	.002	1.550 E-7	1.500 E-5	20	24	.002
Diseases of DNA Double-Strand Break Repair	11	41	14	134	.003	1.650 E-6	1.520 E-4	9	9	5.900 E-4
Signaling by ERBB2 KD Mutants	9	35	7	80	.002	2.770 E-6	2.410 E-4	17	17	.001
PI3K events in ERBB2 signaling	5	22	0	0	.0015	3.990 E-6	3.050 E-4	7	7	4.590 E-4

Supplementary Figure 1. Mutational Co-Occurrence Analysis and Recurrent Mutational Hotspots in CRC Cohort.

Legends: A: Mutual exclusivity and co-occurrence analysis was completed on all variants in the cohort. Twenty paired gene sets were found to be significantly associated. Circus plot details the relationships, with a line between genes identified to have a relationship. B: Multiple variants were found to be recurrent in the cohort. The plot details the top recurring variants, showing how many times each variant was detected in the cohort.

Supplementary Figure 2. Distribution of TMB by Patient Stage:

Legend: Figure details the population distribution of TMB values according to patient stage.

Supplementary Figure 3. Distribution of TMB and MSI status across the cohort.

Legend: Figure shows the distribution of patients with TMB and MSI QC passing samples according to their TMB and MSI status. The threshold for calling TMB-High (TMB-H) was 10 Muts/Mb.

Supplementary figure 4. Median overall survival analysis by pathogenic germline variants

Supplementary Figure 5. Median overall survival by somatic variants

Supplementary figure 6. Recurrence-free survival analysis by pathogenic germline variants

Supplementary figure 7. Recurrence-free survival analysis by somatic variants

Supplementary Methods

Germline Genetic Testing

Full gene-sequencing and deletion/duplication analysis were performed for all genes except where noted below. Full gene-sequencing coverage included coding exons and 10 to 20 base pairs (bp) of adjacent intronic sequence on either side of the coding exons. Additional coverage details for specific genes were as follows.

- *APC*: The 1B promoter region was covered by both sequencing and deletion/duplication analysis and the 1A promoter region was covered by deletion/duplication analysis only. Sequencing analysis for exon 5 included 10 bp of adjacent intronic sequence.
- *ATM*: Sequencing analysis for exons 6, 24, and 43 included 10 bp of adjacent intronic sequence.
- *BMPR1A*: Deletion/duplication analysis included the promoter region.
- *BRCA1*: Sequence analysis included 20 bp of adjacent intronic sequence.
- *BRCA2*: Sequence analysis included 20 bp of adjacent intronic sequence.
- *CDKN2A* (p16INK4a): Analysis supported interpretation of the p14 and p16 proteins.
- *DICER1*: Sequencing analysis for exon 22 included 10 bp of adjacent intronic sequence.
- *EPCAM*: Sequencing analysis was not performed for this gene.
- *FH*: Sequencing analysis for exon 9 included coding sequence and 10 bp of adjacent intronic sequence.
- *GPC3*: Sequencing analysis for exon 3 included coding sequence and 10 bp of adjacent intronic sequence.
- *GREM1*: Promoter region duplication testing only.
- *MAX*: Sequencing analysis for exon 2 included 10 bp of adjacent intronic sequence.
- *MEN1*: Sequencing analysis for exon 2 included 10 bp of adjacent intronic sequence.
- *MET*: Sequencing analysis for exon 12 included 10 bp of adjacent intronic sequence.
- *MITF*: c.952G>A, p.Glu318Lys variant only.
- *MLH1*: Deletion/duplication analysis covered the promoter region. Sequencing analysis for exon 12 included 10 bp of adjacent intronic sequence.
- *MSH2*: Analysis included the exon 1-7 inversion (Boland mutation). Sequencing analysis for exons 2 and 5 included 10 bp of adjacent intronic sequence.
- *MSH3*: Sequencing analysis of the repeat region of exon 1 (5:79950697-79950765) was not performed.
- *MSH6*: Sequencing analysis for exons 7 and 10 included 10 bp of adjacent intronic sequence.
- *NF1*: Sequencing analysis for exons 2, 7, 25, 41, and 48 included 10 bp of adjacent intronic sequence.

- *PHOX2B*: Alanine repeat numbers for the commonly expanded region in exon 3 are not determined.
- *PMS2*: Sequencing analysis for exon 7 included 10 bp of adjacent intronic sequence.
- *POLD1*: Sequencing analysis for exon 22 included 10 bp of adjacent intronic sequence.
- *PTEN*: Deletion/duplication analysis included the promoter region. Sequencing analysis for exon 8 included 10 bp of adjacent intronic sequence.
- *RB1*: Sequencing analysis for exons 15 and 16 included 10 bp of adjacent intronic sequence.
- *RECQL4*: Sequencing analysis for exons 13 and 14 included 10 bp of adjacent intronic sequence.
- *SDHA*: Deletion/duplication analysis was not performed. Exon 14 was not sequenced. Sequencing analysis for exons 6, 7 and 8 included 10 bp of adjacent intronic sequence.
- *SDHC*: Sequencing analysis for exons 2 and 6 included 10 bp of adjacent intronic sequence.
- *TP53*: Deletion/duplication analysis included the promoter region.
- *TSC1*: Sequencing analysis for exon 21 included 10 bp of adjacent intronic sequence.
- *WRN*: Deletion/duplication analysis was not performed for exons 10 and 11. Sequencing analysis for exons 8, 10, and 11 included 10 bp of adjacent intronic sequence.

Interpretation and Classification of Germline Results

Genetic variants were clinically interpreted using Sherloc,[1] a refinement of interpretation guidelines from the American College of Medical Genetics and Genomics and the Association for Molecular Pathology,[2] and classified as pathogenic (P), likely pathogenic (LP), variant(s) of uncertain significance (VUS), likely benign, or benign. P/LP variants (i.e., PGVs) and VUS were reported to clinicians. Genetic test results of a single PGV in a gene associated with autosomal recessive inheritance of cancer risk that carried no recommended clinical management actions for men (*FH* p.Lys477dup, *MUTYH, NTHL1, RECQL4,* or *VHL* p.Arg200Trp) were excluded from additional analyses. However, patients with results of a single PGV in a gene associated with autosomal inheritance of cancer risk that have recommended clinical management actions relevant to men (*BLM*, *RAD50*, *WRN* and *FH* variants other than p.Lys477dup) were included in all analyses.
